# Causality in Scale Space as an Approach to Change Detection

**DOI:** 10.1371/journal.pone.0052253

**Published:** 2012-12-27

**Authors:** Stein Olav Skrøvseth, Johan Gustav Bellika, Fred Godtliebsen

**Affiliations:** 1 Norwegian Centre for Integrated Care and Telemedicine, University Hospital of North Norway, Tromsø, Norway; 2 Department of Computer Science, University of Tromsø, Tromsø, Norway; 3 Department of Mathematics and Statistics, University of Tromsø, Tromsø, Norway; Charité-Universitätsmedizin Berlin, Germany

## Abstract

Kernel density estimation and kernel regression are useful ways to visualize and assess the structure of data. Using these techniques we define a temporal scale space as the vector space spanned by bandwidth and a temporal variable. In this space significance regions that reflect a significant derivative in the kernel smooth similar to those of SiZer (Significant Zero-crossings of derivatives) are indicated. Significance regions are established by hypothesis tests for significant gradient at every point in scale space. Causality is imposed onto the space by restricting to kernels with left-bounded or finite support and shifting kernels forward. We show that these adjustments to the methodology enable early detection of changes in time series constituting live surveillance systems of either count data or unevenly sampled measurements. Warning delays are comparable to standard techniques though comparison shows that other techniques may be better suited for single-scale problems. Our method reliably detects change points even with little to no knowledge about the relevant scale of the problem. Hence the technique will be applicable for a large variety of sources without tailoring. Furthermore this technique enables us to obtain a retrospective reliable interval estimate of the time of a change point rather than a point estimate. We apply the technique to disease outbreak detection based on laboratory confirmed cases for pertussis and influenza as well as blood glucose concentration obtained from patients with diabetes type 1.

## Introduction

When presented with a signal or time series, the identification of change points is often of high importance. Moreover, in live surveillance systems such as disease surveillance, early identification of changes in the system is a core task, where the early warning component invariably is a tradeoff between early detection and the false positive rate through the tuning of model parameters. For weak changes there is also the chance that the method does not detect a real change, a non-detection chance that must be balanced with the other features of the method. In this manuscript we consider two distinct types of processes, point data and unevenly sampled measurements. In the first case, the observations are a series of times 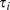 where the interesting feature is the frequency, or density of observations per time unit. For measurements, observations are values 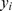 measured at times 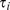, and we would want to detect changes in the underlying process for which 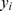 are measurements. The two types of processes lead to two associated methods. Point data are modeled by kernel density estimation, and measurements modeled by kernel regression. The density estimation approach assume observations 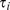 independently sampled from some density 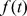 and our goal is to detect changes in this underlying distribution similar to the approach in e.g. [1]. For the regression case, the measurements are values 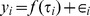 with 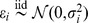, and the interesting aspect is again changes in the underlying value of 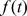. In both cases 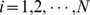 and 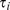 is increasing with 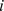, strictly increasing in the measurement case.

Change point detection has a long history in statistics [2] originating in monitoring of industrial processes by way of control charts as pioneered by Shewhart [3]. Later developments on control charts which defined cumulative sums to improve sensitivity, leading to the popular CUSUM algorithm [4]. Recent developments include on-line tools for sequential detection using Bayesian methods [5]. Change point detection has a large variety of applications in a multitude of fields, a few examples include fraud detection [6], climatology [7], and disease surveillance [8]. In the latter case spatio-temporal models are useful for detecting clusters of disease cases [9]. Sequentially detecting changes in live processes is closely related to the problem of detecting change points in complete data, denoted batch detection. The latter was pioneered in Hinkley’s algortihm for detecting change in mean using likelihood ratios [10] or using a Bayesian approach [11].

In statistics causality is often formally defined in terms of directed acyclic graphs as pioneered by Pearl [12] or in the Granger sense [13], while in physics causality is usually imposed on physical theories such that a cause always precedes an effect regardless of the frame of reference. We use causality more in line with the latter definition here. A point data signal is defined as as 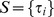 (or the equivalent for measurements 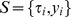). An investigator can choose to analyze the signal retrospectively in a batch analysis, and in this setting causality is unimportant. However, if one wants to detect changes live, the analysis must be strictly causal in the sense that any statistic 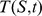 computed at time 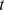 cannot be dependent on future values, 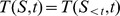 where 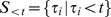 or correspondingly for measurements.

Kernel methods have multiple applications in density estimation and regression [14], classification and pattern recognition methods such as support vector machines [15]. Using kernel density estimation (KDE), the underlying distribution 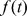 of an observed set of data points 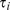 is estimated as
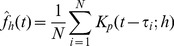
(1)for some kernel 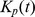 parameterized by a parameter 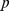 and scale 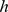 such that 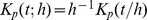. This is the KDE of the distribution. For further details, see, e.g., [14]. The estimate (1) is an asymptotically unbiased estimator of the true density when 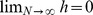 and 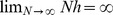. The estimator is unbiased and with variance approaching zero if the true underlying density is well behaved and the kernel is symmetric, bounded and has a finite fourth moment [14]. We allow for different kernels to be used, parameterized by the free parameter 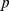. The scaling parameter 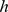, also known as the bandwidth, is a free choice in these models. Note that the KDE definition (1) is not causal in the sense of the preceding paragraph since 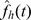 depends on values of 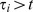 as long as 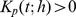 for 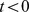.

Choosing a correct bandwidth in (1) is a difficult problem, and indeed may be ill-defined if the true distribution is a mixed distribution with significantly different bandwidths of the components. There are plugin methods to find a bandwidth that are optimal in a specific sense [16], but often the choice of bandwidth remains ambiguous. In some cases not choosing a bandwidth is more appropriate as one is interested in more than one scale. If phenomena happen at different scales, and changes on any scale (or with a large range of scales) are of interest, multiscale approaches are more appropriate. In certain settings the scale is not a priori known, or may change during monitoring, such that using multiple scales is advantageous.

Over the last decade the emergence of scale space methods inspired by similar ideas in image processing have become popular, and a statistical framework has been developed. The SiZer scheme as first described in [17] was developed to assess which structures are significant in noisy settings, either for density analysis or for kernel regression, by analyzing the data on all relevant scales rather than picking out one specific bandwidth. This can provide important information that emerges on different scales and identify structure that might otherwise be missed. In this technique one does not try to estimate the underlying density 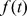, but rather establish whether there is a significant derivative for any time *t* and scale *h*. The space spanned by the two variables is denoted the scale space, and the resulting significance map in scale space gives a quick overview of at what time, and at which scale the significant changes occur. The technique is a quick and visually easily interpretable way to assess important aspects of the data and see important structure. SiZer is not restricted to settings where the predictor is temporal, but we will assume that this is the case for the remainder of the article.

Disease surveillance is an important example of a live processing system where the data are point data, i.e., time of first symptom, confirmed diagnosis or similar. A common way to measure count data is to perform binning, implicitly defining a scale for investigation. Using scale space avoids this scale setting, and adapts automatically to the relevant scale for the problem. Surveillance systems may consist of several independent components, such as laboratory reports, self reporting of symptoms, GP diagnosis reports etc. Combining such heterogenous reports is challenging both from an organizational point of view and for understanding which trends belong to the same incident. For example, a set of symptoms do not map directly to a set of diagnosis. Thus information about the starting point for a trend is useful in combining the sources and understanding the current trends. Moreover, the scale on the outbreak data varies between different infectious agents and even between seasons for seasonal diseases. Thus multiscale monitoring can provide more information compared to single-scale approaches.

Pertussis or whooping cough is a dangerous and highly contagious disease for unvaccinated children, where it is important to identify an outbreak at an early time. Lately, outbreaks in developed regions, such as in California in 2010 have proven that the disease is still a threat to public health, and surveillance of the disease is essential [18]. The seasonal influenza is less severe to most people, but as it affects a large number of people worldwide its consequences are nevertheless large. The regular recurrence makes it useful to report when an outbreak starts, and having estimates of the time an outbreak initiated in each region is important for inference on the progressive spread of the disease. Web based retrievals have become a popular way for the public to be informed early on a prospective outbreak [19]. Models for how communicable diseases spread in a population can augment surveillance system, where recent examples of models include wavefront modelling [20], compartemental models [21] or efficient numerical sampling of Gaussian Markov Random Fields [22].

In our final example we investigate blood glucose data from patients with diabetes mellitus type 1. This is a chronic disease where the pancreas produces insufficient to no amounts of insulin, and most patients must self-administer insulin several times per day. As a part of this self-treatment process, the patients must measure their blood glucose concentration at least three times per day [23], and according to guidelines should keep the concentration between 4–7 mmol/L. It is motivating and useful for the patients to realize trends in their blood glucose, but such trends are often difficult to see due to the high variability of the blood glucose in this group of patients.

In addition to the examples investigated here, the methodology presented is applicable in many setting where early change detection is desirable, or visualization of current trends in real time monitoring within in medical or telemedical systems.

### SiZer

For completeness, we summarize the important aspects of SiZer as formulated in [17] here, and the precise version of the estimators used for the remainder of the paper. For more details, the reader is referred to the original paper or other expositions referenced here.

#### Density estimation

The SiZer (Significant Zero Crossings of derivatives) methodology for nonparametric kernel density estimation [17] abandons the idea of trying to establish a true underlying distribution 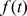 that a set of observations 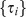 have been drawn from, and reformulates the problem in a hypothesis testing framework. Throughout the two-dimensional scale space spanned by time (*t*) and scale (*h*) we test the hypothesis
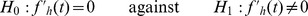
based on 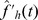 which is the unbiased estimator of the derivative of the kernel smooth (1) with scale parameter 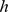. This test is done independently at each location in the scale space, such that a color-coded significance map of significant positive change, significant negative change, or no significant change is established.

The scale space version of the estimator 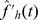 is defined through 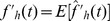 such that 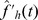 is unbiased at every point in scale space, and one can assume a normal distribution for 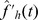, and thus perform the hypothesis testing with a proper estimate of the variance. The precise form of the estimator is trivial,
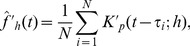
(2)while the estimated standard deviation is simply



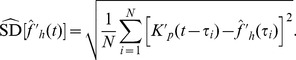
(3)The normality assumption relies on enough observations within the kernel window, and for this purpose an effective sample size is defined,
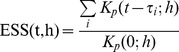
(4)and the normal approximation is considered valid if 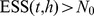 where 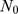 is a choice to make, usually 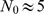.

Since data are reused within a kernel’s support, one must make a correction for multiple testing, where the common choices in SiZer include an assumption of independent tests by introducing the concept of independent blocks at each point in scale space. The number of independent blocks provides an estimate of how many independent tests are performed and is computed as a single number for each scale,
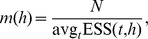
such that each of these blocks of data are considered independent. The simultaneous quantile is thus



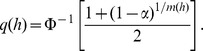



Alternatively one can perform bootstrapping over either *t* or both *t* and *h*, but this option is very computer intensive and usually provides little new information compared to the independent blocks approach which is hence preferred in most cases.

Finally, the results are presented in a scale space diagram, spanned by *t* and *h*, and where each point is color coded according to either significant derivative, not significant derivative, or not enough samples for testing. For further details, see [17], [24], [25].

#### Regression

For regression type problems, with valued observations 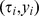, the estimate (1) is replaced by a local linear estimator [14],
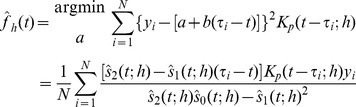
(5)where



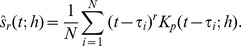



Now the exact same methodology as for density estimation is applied to determine significant changes. The precise form of the estimator for the derivative is, using the unbinned version from [17],
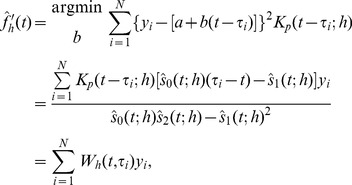
(6)where in the last terms the sum is written as a weighted sum of the observed responses, such that we have defined an equivalent kernel 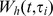:



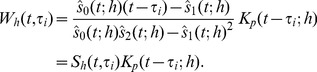
(7)Thus, the derivative estimator is the convolution of the equivalent kernel with the original signal 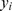, and the equivalent kernel is the original kernel modified by the factor 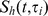. The standard deviation estimator follows directly from the weighted sum in (6) such that [17]

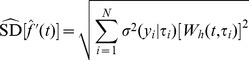
(8)where 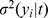 is a smoothed version of the sum of residuals,



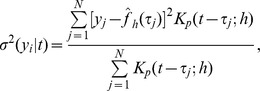
and 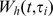 is the weights in the sum of the estimator defined above. The estimated sample size is computed as in the density estimation case. For the estimated sample size it is possible to use the equivalent kernel rather than the original kernel in (4). However, we choose to keep the definition consistent for both the density estimation and regression.

## Methods

Scale space as defined in the previous section cannot be applied meaningfully in live systems where the data set changes as time increases. Then future estimates of the regression curve or density estimates are not meaningful. This is a consequence of scale space not being causal in a physical sense. We define causality as having a strict relationship between an effect and the event that causes the effect such that the event strictly precedes the effect in time [12]. In SiZer-type scale spaces any data point affects both future and past values of the density estimate. This is not a problem if one is doing a retrospective analysis or analysis of non-temporal data. For change detection the goal is to, as quickly as possible, be able to determine if there is a change given the most recent observations available. Indeed, if the chosen kernel has infinite support, as is the case for the Gaussian kernel, causality will not be obeyed since any data point will affect the density estimate for all future and past times. So even though the Gaussian kernel has appreciable features such as a monotone decrease in zero crossings with increasing bandwidth [26], we need to abandon the Gaussian kernel. Kernels that obey causality need to have left-bounded or finite support. Considering causality alone, we could use an asymmetric kernel, such as in [27] where a one-sided kernel has been used to discriminate spike trains. However, asymmetric kernels are no longer asymptotically unbiased estimators, and would lead to different properties of estimators for increases and decreases in the signal. Therefore we consider symmetric kernels exclusively in this paper. Hence we suggest a modified version of the SiZer scheme for early change detection.

The observation that standard kernel density estimation and kernel regression, and hence SiZer, are not causal in the sense that any data point influences the resulting smoothed curve without regard to the time line challenges the application in live systems. A naive approach for KDE is to include a new term in the sum (1) as new data points are revealed, but this would result in a highly discontinuous KDE and a curve that is almost always decreasing except for the places with measure zero where a new datapoint is included. To remedy these issues, we suggest to construct a smoothed curve by using a kernel with finite support, and that is lagged by 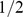 of the support. We define the bandwidth *h* such that the support of the kernel is 2*h* since we will deal exclusively with kernels with finite support. We define the forward KDE (fKDE) as
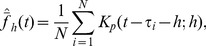
(9)with



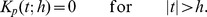



Similarly, for regression estimation, the equivalent kernel 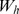 in Eq. (7) is replaced by
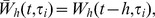
and the regression estimate accordingly. The pointwise variation is



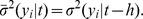



The estimated sample size (ESS) must be corrected using a time shifted kernel, such that the definition (4) still holds replacing 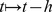. However, the correction for multiple testing must be reconsidered. In c-SiZer, one evaluates a single time point, and as such estimating the number of independent blocks over all prior observations on a single scale is not reasonable. This would imply that the significance tests become increasingly restrictive with time. Hence, we only correct for multiple testing using the data inside the kernel at the time point, i.e., the number of independent blocks is estimated as
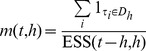
(10)where 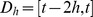 is the region where the past kernel is supported, and the denominator is the number of observations in this region. With this estimate, the significance quantile is as before,
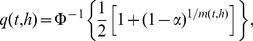
(11)where 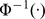 is the inverse standard Gaussian distribution. With these modifications, the standard SiZer computation will yield a significance plot, but where for each bandwidth, the results are progressively shifted by h. We denote the significance plots that arise from this time-shifted kernel density estimate as causal SiZer plots, or c-SiZer plots.

We can define a causal structure in scale space such that an event can only affect future estimators bounded by a causal region that is defined by the kernel’s support. An event in scale space is defined as a location in time, and at zero scale, i.e., when scale space is defined by coordinates 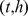, an event is a location 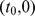. Since the kernel has finite support, the effect of an event can only be measured at a later time, and on scales 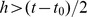. The lines 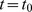 and 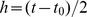 therefore limit the region in scale space where an event at 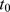 can have an effect. We denote the region inside these lines as the event’s *causal region*. For most kernels significance can never be determined at the edges of the kernel, and we therefore define a more restricted *effective causal region*. The kernel may be effective in an interval 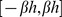 for some parameter 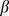. In practice it turns out that it is necessary to distinguish between the lower and upper limits of this region such that the kernel is considered effective in a region 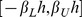 for some 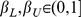. Since a positive change is more likely to arise within the increasing part of the kernel, and vice verse for a negative change, the parameters will be different for the two types of gradient, such that we have four parameters that need to be determined. We use positive change as a reference point, and thus use the notation that 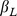 and 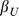 specify the causal region for a positive change, while 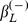 and 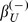 specify the causal region for a negative change. If using a symmetric kernel, which we use exclusively in all examples, the symmetry implies that 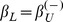 and 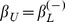. The effective causal region (for a positive change) is delimited by the lines
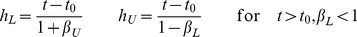
and correspondingly for a negative change. If 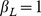, the upper causal region limit is a vertical line, and 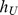 is undefined. The choice of 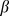
’s is free to make, and should be large enough to accommodate the significant region, while small enough to be effective in the event specification.

**Figure 1:**
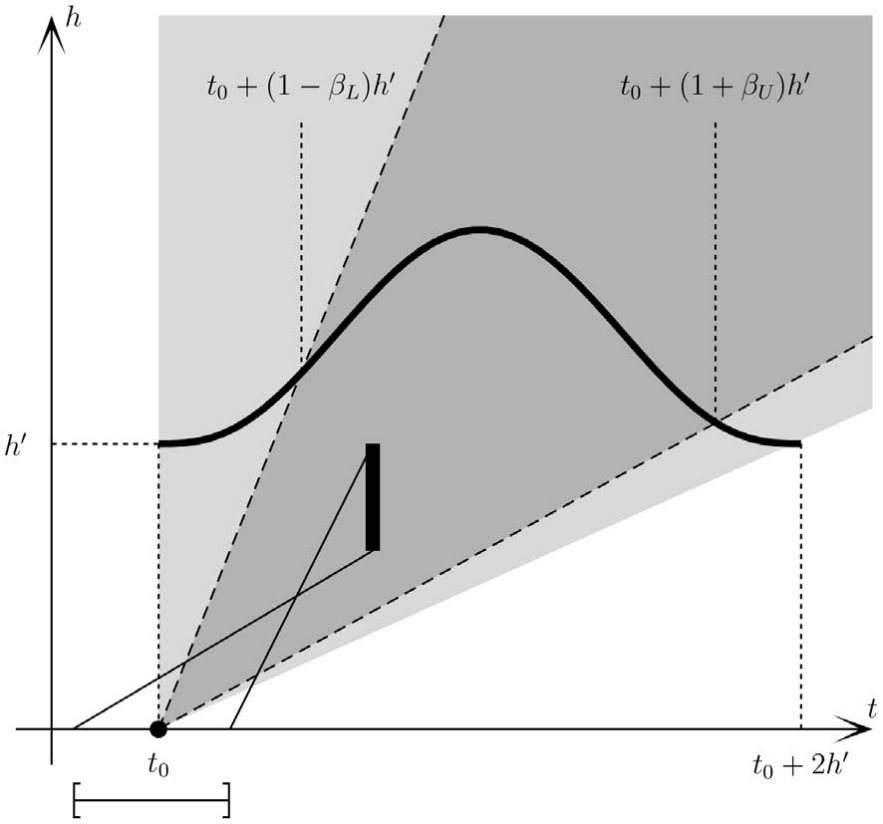
Schematic definition of the causal scale space. An event at time 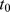 has a causal region in the light gray area and an effective causal region in the dark gray area between the dashed lines. If we detect significance in the interval shown by the vertical thick bar, the past causal region common to all points in this region of scale space infers an originating event in the region indicated by the horizontal bar below the *t*-axis, which encompasses the true event. The definitions of 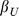 and 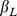 are illustrated by drawing the kernel at one scale 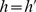. The definition of the effective causal region and hence the 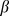
s follow accordingly as described in the text.

The effective causal region is a future causal region from the event at 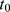, but any region in scale space with 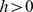 also has a past causal region. If we assume that at some time 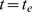 we see a significant change at some scale 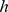, this must arise from an event inside the past causal region. An event at some past time 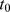 may give rise to a continuous significance area in an interval 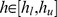 at time 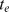. Assuming that the entire continuous area originates from the same event, that event has to be in the past causal region of *all* points in the interval. If we wish to estimate the time 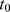 from the information at 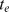, we must find the overlap of all these causal region. Any point 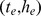 in scale space has a past causal region that gives the time interval 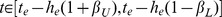. If we consider the interval of significant scales assumed to originate from the same singular event, this event must have happened in the time interval 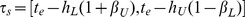. We see that there is a critical value of the ratio 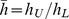, such that if
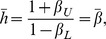
the resulting estimate of the event has zero length, i.e., the event is completely determined. If 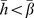, the resulting region in time has a finite length, and we say that the event is specified. If 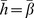, the region is completely specified. Lastly, if 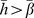, the significance area cannot have resulted from a single event but must be the overlap from several events. We call these unspecified events, since without further information estimating or distinguishing these change points is impossible. In practical applications we have not found this to be a problem. The ideas from this section are summarized in Figure 1.

When analyzing a c-SiZer plot with respect to change detection we ignore all significance areas of the same kind in the future causal region of that first instance, assuming they arise from the same event. However, for a complete picture an analyst could visually examine the full significance map for a more complete understanding of the signal. For example, the magnitude of an instantaneous change will be reflected in how large scales the significance area is translated into, while simple change detection will not provide information about this. The retrospective estimate of the event time automatically reflects the scale at which the change was first detected; a smaller scale means a more precise estimate.

### Kernel

The results may be sensitive to the choice of kernel, and there is a large degree of freedom in choosing the kernel. For both density estimation and regression it is true that the estimators (1) and (5) are asymptotically unbiased for symmetric kernels with finite fourth moment (density estimation) or bounded derivative (regression) [14]. Thus we would prefer kernels that obey these restrictions. Unbiasedness ensures by the central limit theorem that the normality assumption holds. Additionally, we require that the kernel is continuous in value and first derivative, and have finite support. To investigate the effect of the type of kernel, we suggest a family of kernels parameterized by 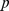, given as
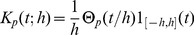
(12)where 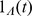 is the indicator function over the interval 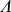, and the defining functions are



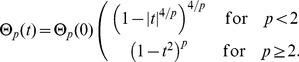
(13)Normalization requires
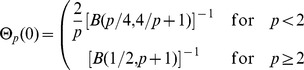
where 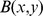 is the Beta function, and 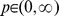. We denote this family the Quartic Family of Kernels (QFK) since the crossover between the two cases, 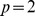 corresponds to the quartic kernel. This definition is motivated by the fact that all these kernels have continuous derivative at 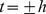, and it smoothly parameterizes distributions ranging from (but excluding) the uniform distribution (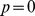) to a Dirac delta distribution (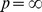). The 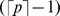 st (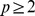) or 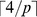
s t (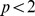) derivative of the kernel is continuous at 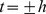. For large and small values of *p*, the kernels becomes essentially discontinuous, and hence the conditions for unbiasedness may no longer hold, thus leading to the normality assumption to be violated. We constructed data with only noise, and confirmed that normality was satisfied within the limits 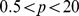, and thus we will use only values in this range for the examples.

An attractive property of this family of kernels is that the entire family is log-concave for all *p*, which guarantees a number of properties such as preservation of this property under convolution [28] and that there is a unique likelihood estimator for the underlying multidimensional distribution [29].

The variance of the kernel is
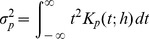


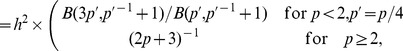
(14)which is seen by identifying the integral with the definition of the Beta function [30]



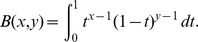



For large *p*, the QFK closely approximates the Gaussian kernel with the same standard deviation, an approximation valid for roughly 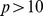.

Some special cases of the kernels, their normalization constant and parameters for the causal region are given in Table 1.

**Table 1:** Examples of kernels in the quartic family.

*p*	Θ_p_(0)	*β_L_*	*β_U_*	Kernel type
0	1/2	1	1	Uniform
1	3315/4096	.677	.820	
4/3	70/81	.663	.828	
2	15/16	.659	.856	Quartic
2.382	1	.615	.818	
3	35/32	.556	.761	Triweight
5	693/512	.438	.624	
10	.541	.298	.449	
∞	∞	0	0	Dirac delta

Normalization constant 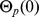 and the parameters of the causal region, 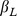 and 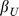 for a selection of values of the kernel parameter *p* in the quartic family of kernels.

### Causal Region

**Figure 2:**
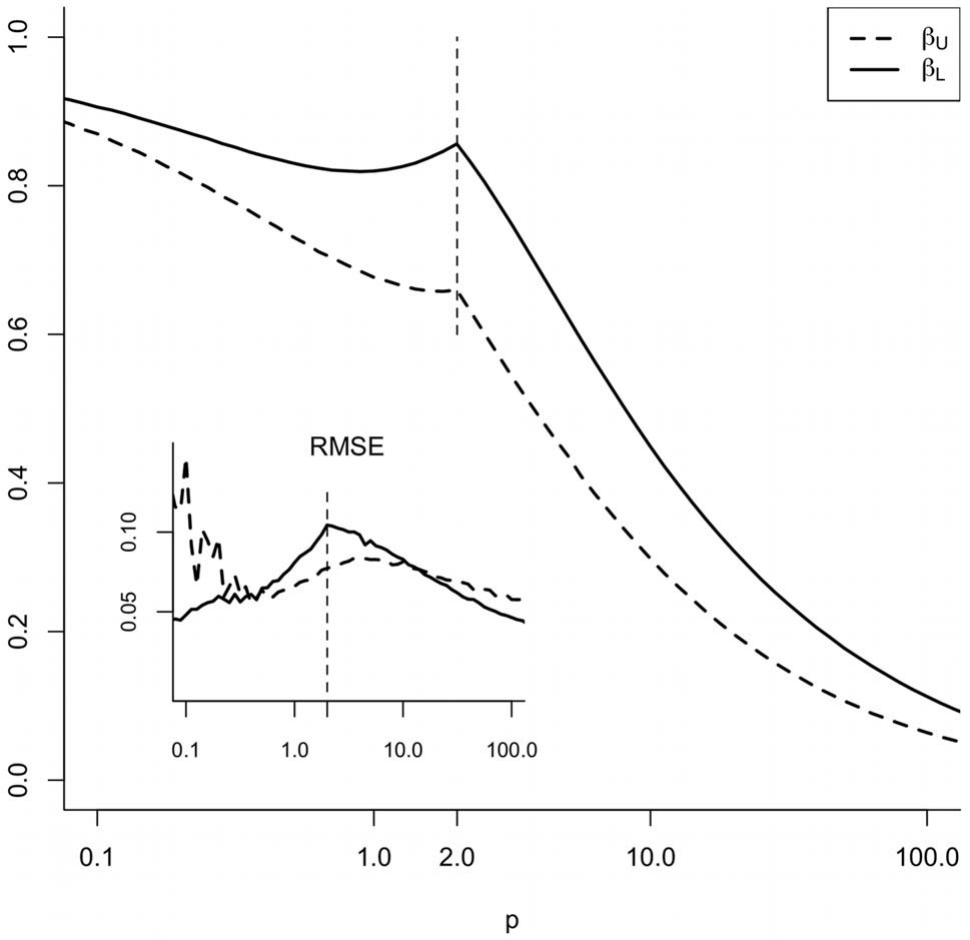
The fitted values of 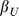 and 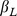 for various *p*. The inset shows the quality of the fit measured by the root mean square error over the same range of 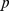. 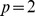 is highlighted by a dashed vertical line.

The parameters of the causal region, 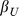 and 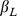 were determined by constructing a deterministic regular series with a significant change at 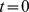. The observations were equally spaced in time with some frequency 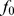 for time 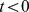 and frequency 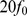 for 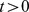. The choice of 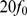 as the discrete jump in frequency for determining the causal region was motivated by making a large jump that creates a distinct causal region, while still keeping the jump within a realistic magnitude. Thus, the large change point at 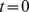 generated a causal region of positive change. We used a linear regression without intercept on the lower and upper ranges of the apparent causal region such that the parameters are determined. The analysis is done for a range of kernel parameters 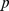 and the values 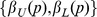 were stored in a dictionary for reference when detecting change points. The resulting values of 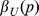 and 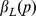 are shown in Figure 2. Note that these estimates were done for a positive change, such that the for a negative change, 
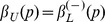
 and 
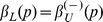
.

### Interval Clustering

At any time point, there may be more than one region of significance, and it might be ambiguous how to merge these to determine or estimate which regions emerge from the same event if they are not all mutually overlapping. For example, it is possible to have three or more regions where number 1 and 2 overlap, number 2 and 3 overlap, but number 1 and 3 do not. Then clearly there are at least two specified events, but it is ambiguous how to merge the events. The clustering of intervals is important to obtain bounds of the events, and we use the following iterative algorithm to merge events.

At any time, a significance region that extends beyond the future causal region of any previously detected event will give rise to a new retrospective estimate of an event. In this way, we will as time increases get a set of estimates 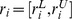 for events of a specific type. Unless they are all mutually exclusive, one would like to merge them to attain more precise information on the retrospective estimates. Assume 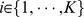 for 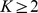. Then, for each pair of regions *i* and *j*, one can compute the overlap between the regions,
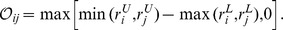



We assume now that at least one off-diagonal element in 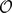 is non-zero. The length of region *i* is denoted 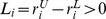, and we define the fraction of the overlap between the regions and their total length through the symmetric matrix
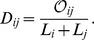



The pair 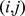 that corresponds to the largest off-diagonal element of 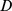 (with ties broken randomly), are merged into a new interval
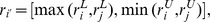
and the procedure is iterated until all 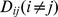 are zero. This procedure reduces the number of identified events as more data becomes available.

### Implementation

c-SiZer and associated methods are implemented in the open-source statistics software package R [31] with some computationally expensive elements implemented in C with an R interface.

### Data Sources

To evaluate the performance of c-SiZer we constructed simulated time series for use in the KDE method. We sampled from a Poisson process with 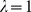 for 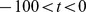, while for 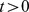 we sampled from a Poisson process with 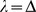 for 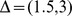. These two values of 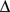 thus indicate a small and large change respectively. The first detection of the change was deduced from the point in the c-SiZer diagram 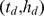 where a positive change was labelled inside the effective causal region of 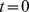 and outside the full causal region of 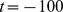, and finding the upper estimate of the event time, 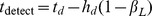. This time will be the first instance where the positive change is detected in the c-SiZer diagram for a live process. For validation we used three comparison methods, a single change point method [10] and CUSUM [4] as implemented in the R-package changepoint [32], and the Bayesian method bcp in the R-package by the same name [33]. For all methods we investigated times up to 100 time units after the change, and concluded that the change point was found if the method estimated a change point within the range of 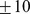 time units from the true change point. For the Bayesian method, we used a threshold for the posterior probability of 0.1, such that the first instance of a posterior probability above the threshold indicated a change, and if there were multiple values above the threshold, the maximal was used. For the three comparison techniques, a binwidth must be set, and three different binwidths *b* of the data were used, 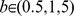. The binning regions were randomly selected such that no prior information about the actual change point was being used.

We used 100 realizations of the Poisson processes, and first detection times were modeled as 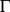 distributed, 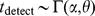. To benchmark the results, we used the same realization scheme as input to the three competing algorithms.

For validation of the false positive rate (FPR) we constructed data sets by randomly sampling 50 numbers from 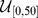, computed the c-SiZer diagram and determined how many of these indicated a positive change outside the full causal region of 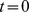. The same method was used to estimate the FPR for the comparison methods. Thus we were able to compare the performance in terms of FPR, true positive rate (TPR), and detection lag for the kernel density estimation approach.

**Figure 3:**
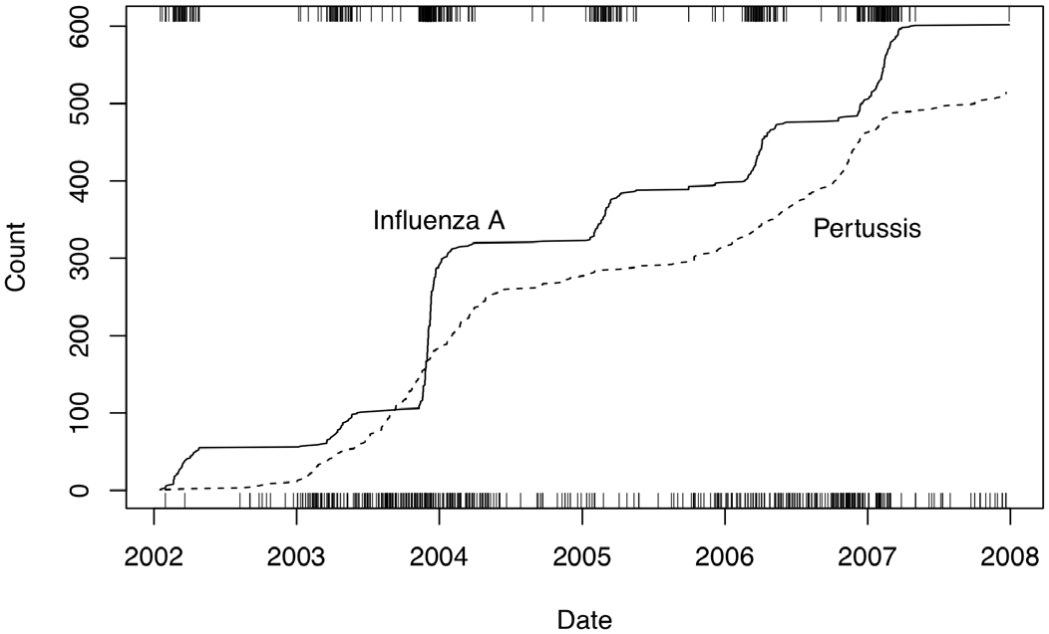
Cumulative plot of confirmed cases of Pertussis and Influenza A 2002–2008. The upper and lower rugs show the individual cases of Influenza A and Pertussis respectively.

As realistic data we used two examples of disease outbreak data and one for self-measurements of blood glucose. Disease outbreak data were obtained from the microbiology laboratory at the University Hospital of North Norway reflecting tests done on samples from the two northernmost counties in Norway, i.e., Troms and Finnmark. These data are adapted for use in the Snow agent system deployed in the Norwegian Health Net, where the current methodology is intended as a key ingredient [34]. These data serve as useful proxies for the real number of cases of the diseases. We have identified all cases of positive tests for the infectious respiratory diseases pertussis and seasonal influenza A in the years 2002 through 2007. Cumulative plots of the raw data for the infectious diseases are shown in Figure 3. For both cases the KDE version of c-SiZer was employed with each confirmed case as a data point.

Lastly we used data gathered from one patient with insulin-regulated diabetes type 1 who was part of a larger cohort of patients in an exploratory study on a mobile phone application for this patient group [35]. The patient used self-measured blood glucose as part of the treatment process, and we used the data for self-measured blood glucose as input for the regression version of c-SiZer.

## Results

### Simulated Data

**Figure 4:**
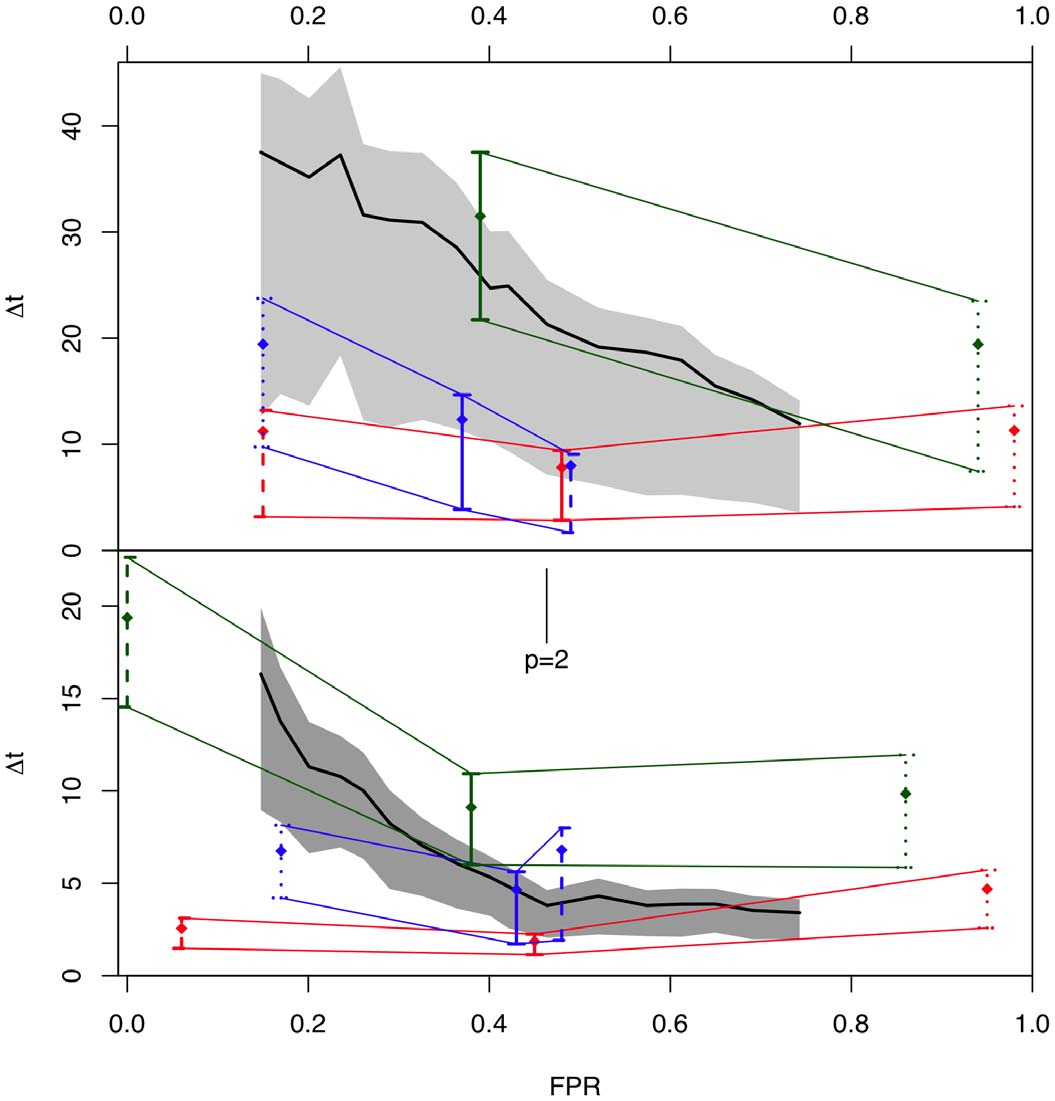
Detection of change in constructed time series. The detection time for a small change (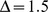, top panel) and large change (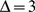, bottom panel) versus false positive rate for the c-SiZer algorithm with *p* in the range 20 (left in plot) through 0.5 (right in plot). The mean detection time is shown as black line for the two cases, while the surrounding shaded regions show the 30/70 percent quantiles for the fitted 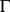 distribution. The FPR for 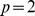 is indicated. The colored lines show the corresponding values for the comparison algorithms cpt (red), bayes (blue), and cusum(green) for three different binwidths, 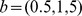 with (dashed, full, dotted) lines. Note that for cusum, the 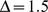 change is never detected using binwidth 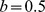.

**Figure 5:**
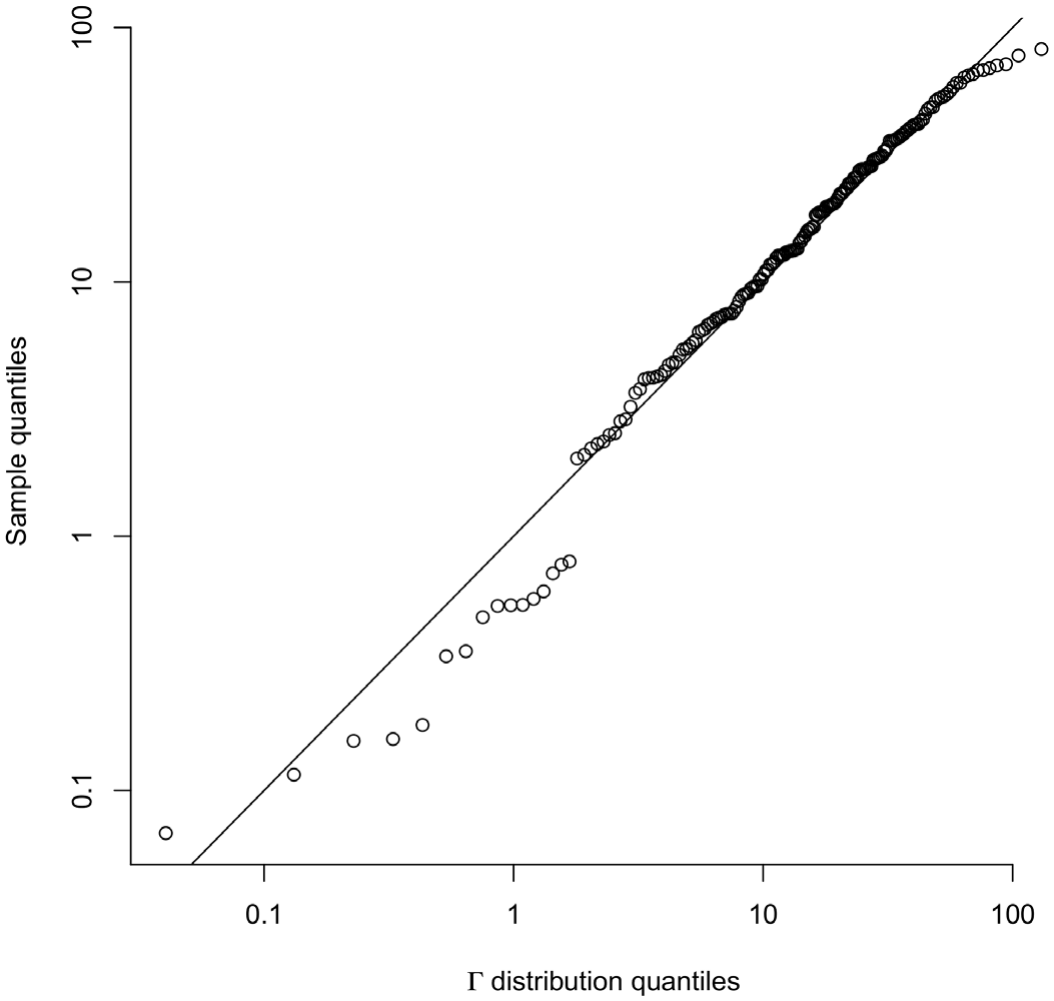
Fit of observed lags to the 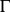 distribution. Q-Q plot for 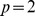 versus the fitted 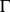 distribution. The p-value for a Kolmogorov-Smirnov test in this case is 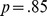.

The parameters 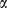 and 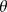 in the 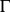 distribution model were fitted to the realizations of waiting times, and found the mean 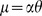 which is plotted along with upper and lower 30% quantiles of the fitted Gamma distribution in Figure 4. Corresponding values for the three comparison methods are also shown. The validity of the 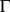 distribution was confirmed by doing Kolmogorov-Smirnov tests and Q-Q plots. The Q-Q plot for 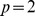 is shown in Figure 5, and the 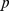-values for the Kolmogorov-Smirnov tests are above.01 for all relevant values of *p*, most above.1, and range up to.9. In Figure 4 we show the relation between the detection time and false positive rate for c-SiZer for the range of *p* and the chosen values of 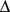 along with results for the comparison methods using three different binning widths. In Table 2 we show the TPR for the comparison methods for chosen binning and 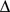. c-SiZer eventually detected all changes, thus having a TPR of 1.

**Table 2:** True positive rate for detection algorithms.

*b*	Δ	cpt	bayes	cusum
0.5	1.5	.68	.45	.0
0.5	3	1.0	.97	1.0
1	1.5	.83	.48	.67
1	3	1.0	.98	1.0
5	1.5	.82	.48	.75
5	3	1.0	.98	1.0

Number of times each algorithm eventually detected a change for the combinations of magnitude of change Δ and binwidth *b*. Note that for cSiZer all changes were found, giving a true positive rate of 1.

### Real Data

**Figure 6:**
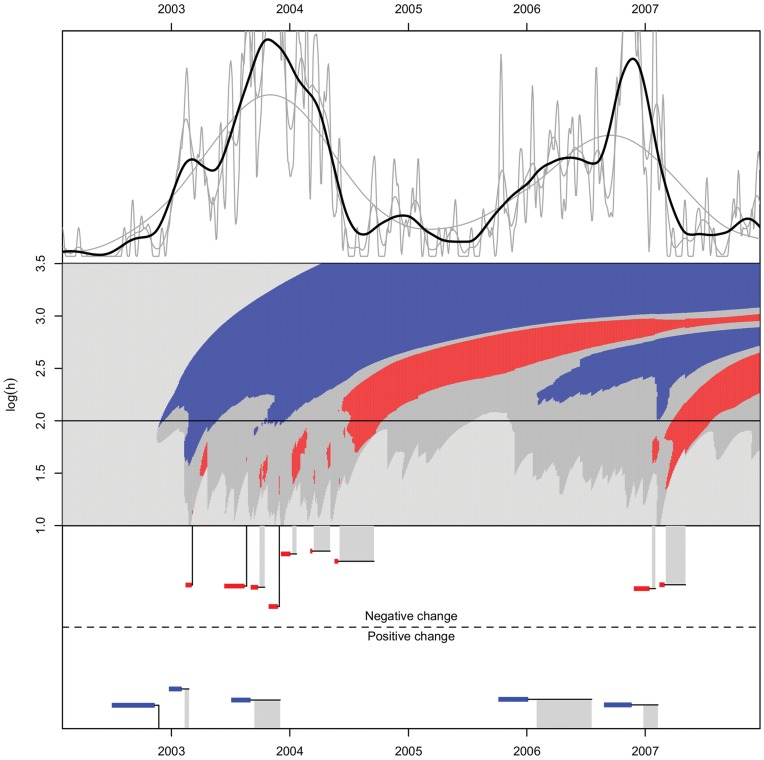
c-SiZer KDE analysis of incidence of Pertussis in North Norway in the period 2002–2007. *Upper panel*: Kernel density estimates of confirmed cases of pertussis with varying bandwidths, 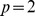. No vertical scale is indicated since the live signal cannot be consistently normalized. *Middle panel*: The c-SiZer plot, where in shades of gray from light to dark (color online) indicate 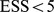, no significant derivative, significant decrease (red), and significant increase (blue). No significant derivative means that the null hypothesis is not rejected. The horizontal line corresponds to the bandwidth used in the black line of the kernel density plot. Logarithm is taken base 10, *h* has units days in this and subsequent plots. The underlying data have a resolution of one day here and in Figure 7. *Lower panel*: Indication of the detected changes with the specification of the event interval after clustering. The horizontal colored lines indicate the inferred region for the change point, while the connected vertical line is the detection point. Vertical gray bars, show that the intervals are clustered and indicate the range in which the detections are made. For clarity the height of the change indicators in the lower panel are jittered.

The kernel density estimate for pertussis and the corresponding significance plot are shown in Figure 6. In this and all subsequent examples the kernel (12) with 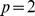 is used. Also shown are the identified change points after clustering. We clearly see two important outbreaks in the period, starting late 2002 and mid 2005, and there is much important structure in the data. From an early disease detection standpoint it is obviously important to give warnings both at an early phase, and also at a later point if there are further significant increases or decreases. Note that causality emerges in the c-SiZer plot, as significance regions deflect to the right, into the respective events’ causal regions. Note that the initial increase at top left is likely to emerge because the 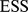 abruptly raises above the threshold at one time point, giving significance at many more scales than would be justified from the efficient causal region of the event. This effect is likely to arise in all cases using KDE from the start of the recording and must be accounted for, e.g., by excluding the full causal region of the start of recording in an analysis.

**Figure 7:**
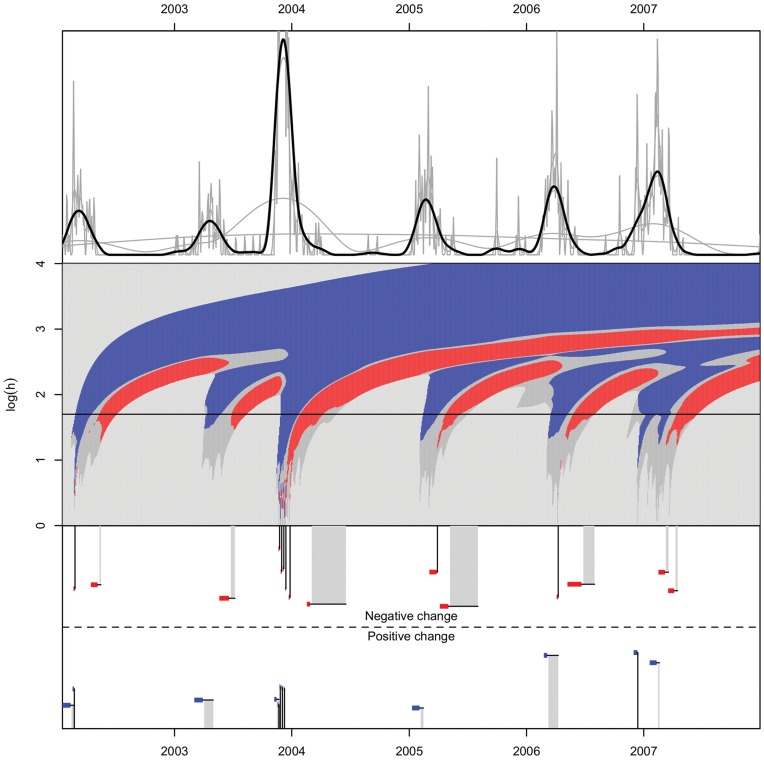
c-SiZer KDE analysis of the number of confirmed laboratory cases of influenza A in North Norway in the period 2002–2007. The structure of the figure is as in Figure 6. The regular recurrence of the influenza is clearly shown. Also note that the large outbreak in the 2003/2004 season give significance on details in the plot due to test patterns in the laboratory and the high volume of data here.

The resulting c-SiZer analysis for influenza A is shown in Figure 7. Every outbreak is identified with a proper retrospective estimate of the outbreak time. Note that the large outbreak in the season 2004/2005 have small time frames of the retrospective outbreak (and correspondingly detection at small scales), which indicated a high transmissibility of that year’s outbreak. This was again reflected in the large outbreak that subsequently happened.

**Figure 8:**
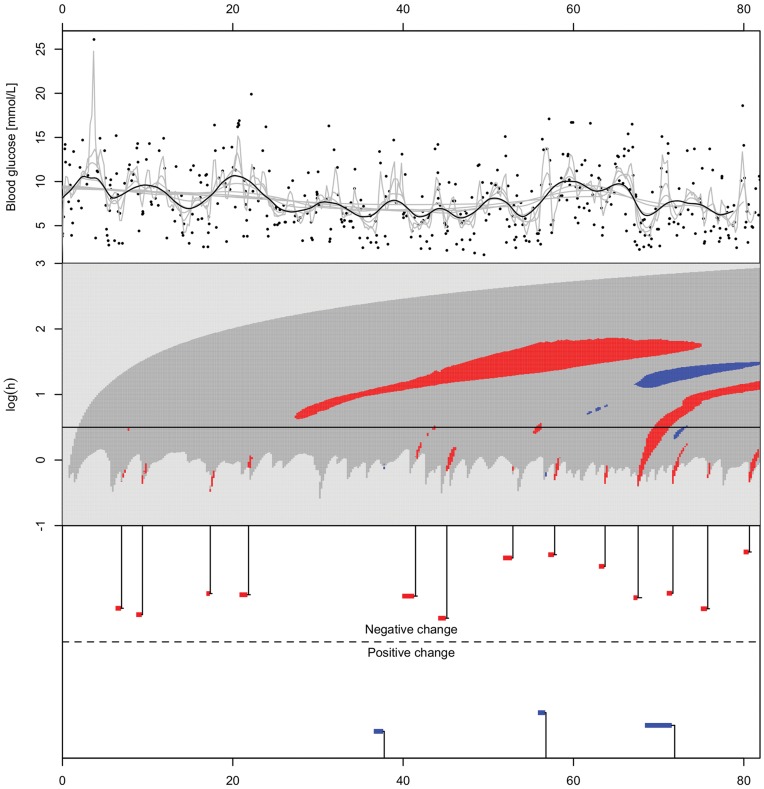
Blood glucose measurements for one patient with type 1 diabetes over 82 days, along with c-SiZer regression analysis. The structure of the figure is as in Figs 6 and 7. The raw data are self-measured blood glucose with time stamp resolution of one second as reported by the blood glucose meter.

Finally, the c-SiZer analysis for one patient’s self recorded blood glucose values for three months is shown in Figure 8. We see that important information that could be valuable for the patient emerges on different scales as time progresses.

## Discussion

We have shown how imposing causality on the scale space constructed by SiZer-type plots gives a way to early detect significant changes in a temporal data set with observations, and by adjusting the kernel used one can achieve an appropriate tradeoff between false positive rate and early detection. In particular, the technique has low false positive rate and early detection time compared to the popular CUSUM technique. We have demonstrated the technique for both point data and regression type problems.

The causal structure carries the added benefit that it makes it possible to retrospectively infer an estimated range for the change points in a statistically meaningful way. While often not of critical importance, this may be of value in many applications. In particular when collecting data from heterogenous sources comparing the initiating point from these sources can add evidence to hypotheses of how and when the change originated, disease surveillance being an important example.

In both regression and point data problems, the issue of scale is important, and is often reflected in a binning for temporal data. Binning automatically sets a scale that is believed to be relevant for the data. Hence, structures on other scales are missed. Using a scale space approach eliminates this issue since all relevant scales automatically are scanned.

When comparing to other methods, c-SiZer is not uniquely better in all respects, and e.g. the Bayesian method is preferable for the case of a small change for all binwidths investigated. However, this reflects a prior knowledge of the scale of operation, and often that is not the case. Also, a signal may consist of both small and large scale changes, such that any one parameter setting for a method is not applicable. c-SiZer for a large part avoids this choice depending only on a single kernel parameter. Notably, c-SiZer is the only investigated method where the true positive rate was one throughout, i.e., all changes were eventually detected. Finally, c-SiZer provides a significance plot that, if interpreted properly, gives rich insight into the structure of the data set beyond a simple assessment of the change points. Hence just a comparison with other change point methods does not convey the method’s full potential since the visual structure inherited from SiZer significance maps is still available. In particular, when studying live data such as the blood glucose data, a snapshot of current significant derivatives on different scales gives more information to the user than simply investigating a single scale, and irrespective of the change point analysis.

The kernels investigated here are obviously not unique, and other kernels can easily be conceived. In particular, the restriction of the kernel to be symmetric may not be necessary, dependent on the specific setting. For preservation of causality, the kernel’s support does not need to be finite, but left-bounded, though that would loose the structure necessary for interval change point estimation. Using a non-symmetric kernel leads to different detection times and false positive rates for positive and negative changes, i.e., the positive and negative tests are at different points in the diagram of Figure 4. Moreover, an asymmetric kernel means that the kernel density estimate no longer necessarily is an unbiased estimator of an underlying density or regression curve, so the results must be interpreted with care in these cases.

The method described in this paper relies heavily on the assumption of normally distributed residuals outside the change regions. Ideally, normality follows from the central limit theorem provided a well-behaved kernel. However, for kernels that are not sufficiently smooth this assumption may no longer hold, and should be carefully assessed if other kernels are being applied.

For both the examples of infectious disease outbreak detection the method can be useful for detecting changes and outbreaks in itself or as part of a larger framework for disease surveillance. For diabetes patients, both type 1 and 2, c-SiZer can be a useful tool for the patients in their self-treatment process such that they are warned or informed of changes in the blood glucose pattern, and when the cause of the change happened. If significant changes in the patient’s lifestyle occur, the blood glucose pattern may also change, and alerting the patient of the changes along with the time of likely cause will help the patient identify the cause of the pattern and take suitable action to correct the blood glucose levels, if appropriate. Notably, inferring which changes are significant or not is difficult based on the raw values in the upper part of Figure 8. The full c-SiZer plot provides much more information on the signal, which in turn are summarized in the change point estimation.

Note that in Figure 6 there are overlapping regions of positive and negative change. This may occur in any data set, and can arise in two different ways. Firstly, the regions are simply estimated ranges of a change point, and thus there may be one positive and one negative change point at different times within the respective estimates. Secondly the change points may indeed be overlapping in time but at different scales. Thus there may be a significant negative change on one scale simultaneous with a significant positive change at another scale. If the reason is considered important to the problem at hand, a careful investigation of the c-SiZer plot would reveal the likely cause. For the case of the overlapping regions in Figure 6, it is clear from the c-SiZer plot that the overlapping regions happen where the negative changes are on much smaller scales than the positive changes, thus reflecting small scale variation contained within a larger positive trend.

In all examples here we have used raw data, but correcting the data for, e.g., seasonal or other periodic variation is easily conceivable depending on the usage. For example, diabetes patients’ blood glucose may have significant variation through the day and correcting for these will alert them to trends above these variations, and possibly give earlier alerts by reducing noise.
